# An Improved Nanoemulsion Formulation Containing Kojic Monooleate: Optimization, Characterization and In Vitro Studies

**DOI:** 10.3390/molecules25112616

**Published:** 2020-06-04

**Authors:** Muhammad Azimuddin Roselan, Siti Efliza Ashari, Nur Hana Faujan, Siti Munirah Mohd Faudzi, Rosfarizan Mohamad

**Affiliations:** 1 Department of Chemistry, Faculty of Science, Universiti Putra Malaysia, Serdang 43400, Selangor, Malaysia; azim96.kim@gmail.com (M.A.R.); nurhana@upm.edu.my (N.H.F.); sitimunirah@upm.edu.my (S.M.M.F.); 2 Integrated Chemical BioPhysics Research, Faculty of Science, Universiti Putra Malaysia, Serdang 43400, Selangor, Malaysia; 3 Centre of Foundation Studies for Agricultural Sciences, Universiti Putra Malaysia, Serdang 43400, Selangor, Malaysia; 4 Laboratory of Natural Products, Institute of Bioscience, Universiti Putra Malaysia, Serdang 43400, Selangor, Malaysia; 5 Department of Bioprocess Technology, Faculty of Biotechnology, Universiti Putra Malaysia, Serdang 43400, Selangor, Malaysia; farizan@upm.edu.my

**Keywords:** response surface methodology, hyperpigmentation, nanoemulsion, kojic monooleate, optimization, formulation, cytotoxicity, tyrosinase inhibitory activity, in vitro

## Abstract

Tyrosinase inhibitors have become increasingly important targets for hyperpigmentation disease treatment. Kojic monooleate (KMO), synthesized from the esterification of kojic acid and oleic acid, has shown a better depigmenting effect than kojic acid. In this study, the process parameters include the speed of high shear, the time of high shear and the speed of the stirrer in the production of nanoemulsion containing KMO was optimized using Response Surface Methodology (RSM), as well as evaluated in terms of its physicochemical properties, safety and efficacy. The optimized condition for the formulation of KMO nanoemulsion was 8.04 min (time of high shear), 4905.42 rpm (speed of high shear), and 271.77 rpm (speed of stirrer), which resulted in a droplet size of 103.97 nm. An analysis of variance (ANOVA) showed that the fitness of the quadratic polynomial fit the experimental data with large *F*-values (148.79) and small *p*-values (*p* < 0.0001) and an insignificant lack of fit. The optimized nanoemulsion containing KMO with a pH value of 5.75, showed a high conductivity value (3.98 mS/cm), which indicated that the nanoemulsion containing KMO was identified as an oil-in-water type of nanoemulsion. The nanoemulsion remains stable (no phase separation) under a centrifugation test and displays accelerated stability during storage at 4, 25 and 45 °C over 90 days. The cytotoxicity assay showed that the optimized nanoemulsion was less toxic, with a 50% inhibition of cell viability (IC_50_) > 500 μg/mL, and that it can inhibit 67.12% of tyrosinase activity. This study reveals that KMO is a promising candidate for the development of a safe cosmetic agent to prevent hyperpigmentation.

## 1. Introduction

Skin hyperpigmentation disease occurs when there is an abnormal production of melanin synthesized in melanocyte. This can be caused by an excess of melanin level in the skin through several factors such as UV radiation, radicals, inflammatory mediators and hormones [1]. Melanin is a skin pigment, derived from tyrosine during the melanogenesis process, which determines the color of mammalian skin, eyes and hair [2]. There are two types of melanin: eumelanin and pheomelanin. The black-brown eumelanin is involved in black or brown hair and dark skin, whereas the red-yellow pheomelanin is responsible for red hair [3]. In humans, tyrosinase is involved in the biosynthesis of melanin and results in various skin disorders such as hyperpigmentation, melisma, malign melanoma and skin-aging processes [4,5]. General melanin biosynthesis involves three stages. The first stage is the hydroxylation of l-tyrosine to 3,4-dihydroxy phenyl l-alanine (L-DOPA), followed by stage two, which is the oxidation of L-DOPA to DOPA–quinone and, lastly, stage 3, which is the non-enzymatic polymerization of DOPA–quinone to melanin [6,7]. The first and second stage are catalyzed by tyrosinase.

Tyrosinase has been considered as an important enzyme for the development of therapeutic agents for hyperpigmentation diseases in the cosmeceuticals field [8]. The inhibition of tyrosinase can greatly affect the production of melanin and solve hyperpigmentation [9]. In order to achieve this, extensive research involving both natural and synthetic skin-whitening agents, has been carried out.

Kojic acid (KA) is one of the most-used skin-whitening agents. It is an antibiotic produced by many species of *Aspergillus*, *Acetobactor,* and *Penicillum* through an aerobic process [10]. In addition, KA is commonly used in the food industry to prevent the browning effect, as well being used in cosmetic preparations as a skin-lightening or bleaching agent due to its ability to inhibit tyrosinase [11,12]. However, KA was described as an unstable compound [10,13] that is sensitive to light and heat [14]. The effectiveness of KA as a whitening agent is reduced when exposed to sun or air [14]. Furthermore, this natural compound tends to oxidize over time. There are also several concerns regarding its toxicity, mutagenicity and carcinogenicity, which spurred Japanese officials to ban the use of this compound in any skin-related treatment [12,15]. Therefore, modifications by synthesizing KA derivatives have been made in order to seek stable, non-toxic and effective whitening agents.

The esterification of KA and oleic acid (OA) is one of the possible methods for producing KA ester. KA ester has been found to have excellent effects, such as skin-whitening and an anti-suntan effect [16]. KA derivatives were also determined to be fifteen times more stable than their source (KA) in terms of their storage stability, compatibility and also oil solubility [11]. Lajis et al. [9] reported that KA esters were safe and nontoxic depigmenting agents that showed satisfactory inhibitory effects on tyrosinase activity, as determined in B16F1 melanoma cells. Kojic monooleate (KMO), which is a palm-based fatty acid derivative of KA, was found to have a significant effect in tyrosinase inhibition when compared with kojic monolaurate and kojic monopalmitate [17]. This shows that KA esters, specifically KMO, can be used as skin-whitening agents in order to inhibit tyrosinase and treat hyperpigmentation.

To inhibit tyrosinase, the active ingredient (skin-whitening agent) should be able to penetrate the stratum corneum (SC) and epidermis by incorporating the active ingredient into a nanoemulsion system. Nanoemulsion is made up of two immiscible liquids or phases, where one liquid is dispersed into another liquid in the presence of a surfactant, in order to lower the surface tension between the two fluids, and to obtain a droplet size ranging from 20 to 500 nm [18,19,20]. Researchers have also found that nanoemulsion is an emulsion system with droplet sizes ranging from 20 to 200 nm [21]. In recent years, nanoemulsion has become a popular potential vehicle in the cosmeceutical field due to its small-sized droplets with high surface areas, which can effectively transport and deliver skin-whitening agents to the targeted site [19,22]. Moreover, due to its small droplet size, nanoemulsion possess excellent stability against sedimentation and creaming [20]. The need for surfactant in nanoemulsion is low (5–10%) compared to microemulsion (20–25%), making nanoemulsion less of an irritant to the skin and more preferable as a carrier in the cosmeceuticals field [23,24]. The nanoemulsion formulation provides the rapid penetration of the active ingredients through the skin due to the large surface area of its droplets. It has been found that nanoemulsion can even penetrate through rough skin easily. Based on the good sensorial properties and biophysical properties, nanoemulsion has been determined as a good delivery system in topical applications. There are various reported methods which support the administration of a nanoemulsion formulation through topical applications [25,26,27,28,29]. KMO is a poor water-soluble compound, and thus nanoemulsion is considered to be the most advanced nanoparticulate system for cosmeceutical uses, because it contains submicron-sized droplets varying from 20–200 nm, which can easily penetrate into deep skin cells [30], thus solving hyperpigmentation. Furthermore, as well as its uses in the cosmeceuticals field or industry, the properties of nanoemulsion, such as its groups of dispersed droplets and spherical shape, allow it to be used in other industries, such as food, chemical and pesticide industries [24,31,32].

The process parameters involved in producing nanoemulsion should be optimized in order to obtain the desired droplet size. The conventional method (one factor at a time) is time-consuming and costly [33]. The desired droplet size of nanoemulsion can be optimized by using Response Surface Methodology (RSM). RSM is the most popular optimization method used in recent years [34]. It is a collection of statistical and mathematical techniques, which are useful for developing, improving, and also optimizing processes where several independent variables influence a response or a few responses of interest [34]. RSM is capable of assessing the interaction and relationship between independent variables (factors) and dependent variables (responses) in decreasing the number of experimental runs, with a desirable result obtained in optimum conditions [35,36]. Therefore, RSM was employed to study the interaction between three variables (the time of high shear, the speed of high shear, and the speed of the stirrer) with droplet size as the response.

The aim of the present research is to optimize the parameters (the time of high shear, the speed of high shear and the speed of the stirrer) in the production of nanoemulsion containing KMO (previously developed by Syed Azhar et al. [17]), as well as to evaluate the physicochemical properties of optimized nanoemulsion. The efficacy and safety of optimized nanoemulsion containing KMO are also studied, using cytotoxicity and a tyrosinase inhibition assay.

## 2. Results and Discussion

### 2.1. Fitting the Model

The mean droplet size of the nanoemulsion obtained experimentally, according to the central composite design (CCD), is tabulated in Table 1.

The table shows that the minimum droplet size obtained was 103.16 nm (run no. 13). The actual droplet size values were mostly in good agreement with the predicted values. The final equation to predict the droplet size in terms of coded factors for the second-order polynomial is shown in Equation (1):

Y = 111.82 + 7.32A − 3.78B − 3.04C − 10.17AB + 4.58AC + 0.038BC + 5.41A^2^ + 3.00B^2^ + 5.22C^2^ (1)

where A, B, and C represent the values of the time of high shear, the speed of high shear, and the speed of the stirrer, respectively.

**Figure 1:**
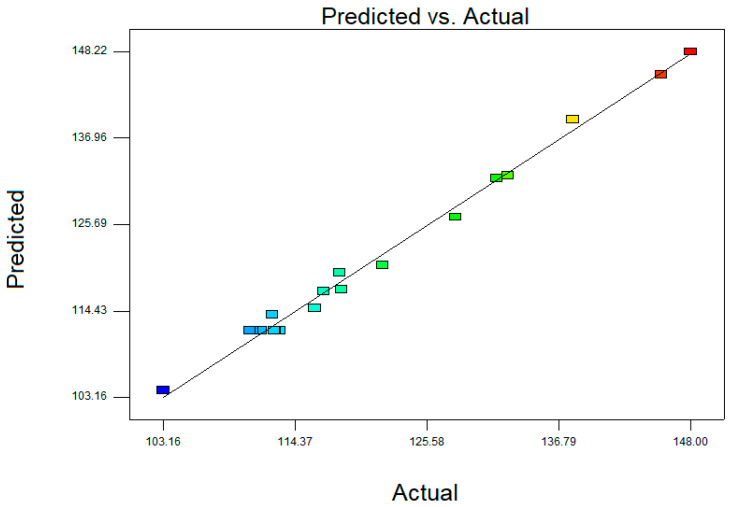
Scatter plot of predicted droplet size values versus actual droplet size values from three-factor central composite design (CCD).

The predicted droplet size values derived from the model versus the actual droplet size values obtained from the experimental data shows that the model was successful in capturing the correlation between all the independent variables, with R^2^ = 0.9933 (Figure 1).

ANOVA results for the effects of all independent variables were implemented using Design-Expert^®^ software (version 7.1.5) in order to investigate the suitability and also the significance of the final model (Table 2).

A large *F*-value and small *p*-value would mean that independent variables have a significant and larges impact on the respective response [37,38,39,40]. The final model was found to be significant, with a large *F*-value (148.79) and a small *p*-value (<0.0001), and the lack of fit was insignificant in terms of the response (droplet size). All terms were found to be significant, except for the interaction terms between the speed of high shear (B) and the speed of the stirrer (C), (BC). The model is reliable enough to predict a variation in droplet size. The adequacy and validity of the mathematical equations were evaluated by regression coefficient results for the final reduced model (Table 3). It was found that the predicted *R*^2^ (0.9494) was in reasonable agreement with the adjusted *R*^2^ (0.9866). In addition, the CV values were less than 10 and adequate precision values were greater than four; all these statistical parameters show the reliability of the models [37,41].

### 2.2. Response Surface Analysis

**Figure 2:**
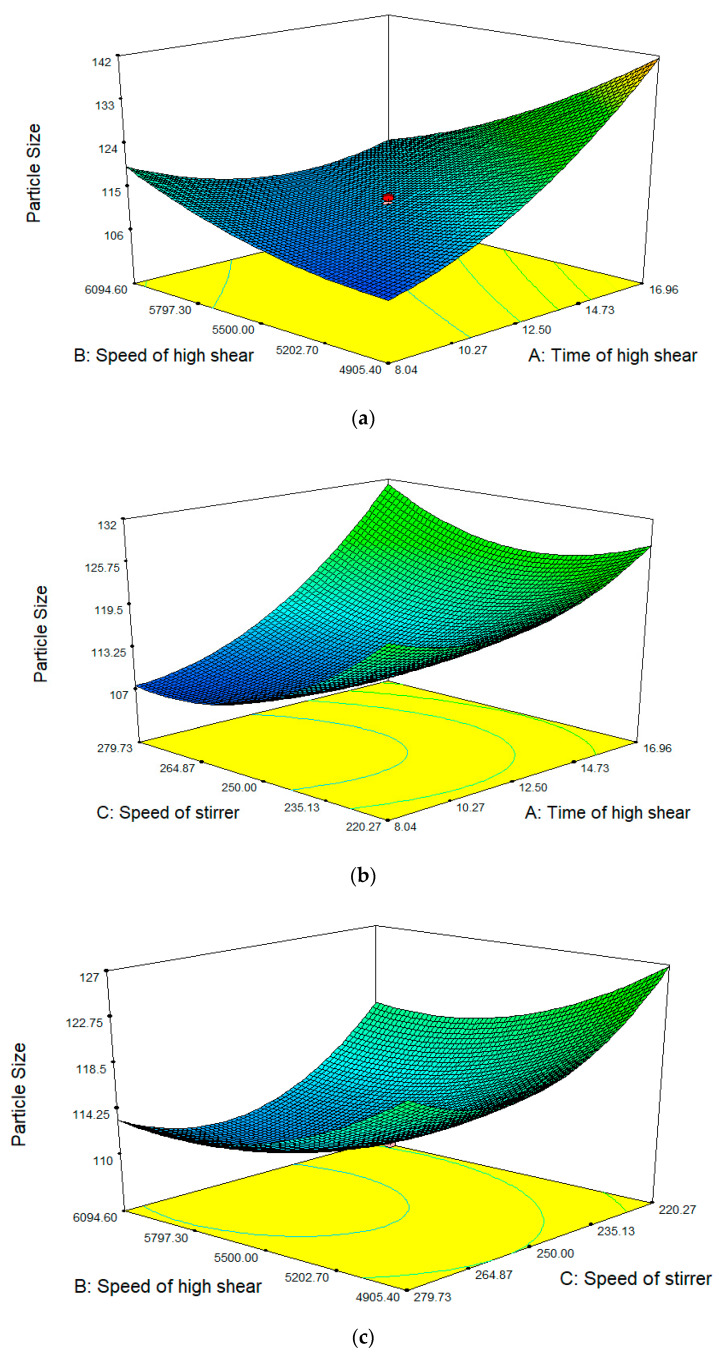
Response surface showing the effect of (**a**) time of high shear and speed of high shear; (**b**) time of high shear and speed of stirrer; (**c**) speed of high shear and speed of stirrer.

For cosmeceutical purposes, nanoemulsions are preferable because they have a good spreadability and can facilitate the penetration of active ingredients into the skin [42]. Nanoemulsion creates a large surface-to-volume ratio of emulsion particles that make contact with the skin, thus enabling more active ingredients to make contact with the skin via a surface-to-surface interaction between the emulsion and the skin. Therefore, the main factor that has to be considered for this design is the minimum droplet size. For the optimization of a nanoemulsion containing KMO, response surface analyses were plotted in three-dimensional model graphs (Figure 2) generated using the Design-Expert^®^ software (version 7.1.5).

Table 2. demonstrates that, as the time of high shear increases, the droplet size of nanoemulsion also increases. In high shear homogenization, the nanoemulsion containing KMO was forced to go through the small holes surrounding the probe, which caused the droplet to break down, forming smaller particles. However, in this study, a longer high shear time led to an increasing droplet size, which might be attributed to the fact that this condition also promoted a higher rate of collision between droplets. This promotes the aggregation of small droplets into larger droplets, as reported by Einhorn-Stoll et al. [43] and Rebolleda et al. [44], who observed that high shear gave the smallest droplet size, but exhibited rapid destabilization after a few hours. This finding opposes Li et al. [45], who reported that droplet size decreases with an increasing high shear time; however, the maximum time of high shear that was used in their study was only 3 min.

On the other hand, the droplet size of the nanoemulsion increases at 8.04 min (time of high shear) with the increasing speed of high shear. However, at longer times of high shear, the droplet size of the nanoemulsion decreases with the increasing speed of high shear. It was also found that, at any stirrer speed, the droplet size of the nanoemulsion decreases when the speed of high shear increases. The high speed of stirring diminished the droplet size of the nanoemulsion due to the turbulent flow and hydrodynamic shear stress [45]. The increasing speed of high shear means that the shear force given to the droplets became more powerful, thus leading to droplets being broken down into smaller droplets. The increasing speed of high shear also led to an increase in the temperature, which then caused a decrease in the viscosity of the dispersion system, resulting in smaller droplet sizes.

As the speed of the stirrer increases, the droplet size also decreases. This homogenization technique is a spontaneous method using an overhead stirrer. In this homogenization technique, the nanoemulsion was only subjected to a simple stirring process. The droplets are not subjected to a strong force, which could break them down significantly. However, stirring helps to homogenize and increase the spreadability of the nanoemulsion. This results in a small droplet size for the nanoemulsion when the speed of the stirrer increases.

### 2.3. Verification of Model

The verification of the final mathematical model was done in order to insure the adequacy of the predicted response values in the validation set. To test the sufficiency of the final obtained model, certain randomized formulations were obtained by point prediction using the Design-Expert^®^ software (version 7.1.5). Independent variables in the verification set were not found among twenty main runs but were found in the defined ranges. The actual and predicted droplet sizes of the validation set are tabulated in Table 4. Five different formulations were selected. The residual standard error value was calculated to observe any significant difference between the actual and predicted droplet size, as shown in Equation (2):

(2) $$\mathrm{Residual}\;\mathrm{Standard}\;\mathrm{Error}(\%)=\frac{\mathrm{Actual}\;\mathrm{Value}-\mathrm{Predicted}\;\mathrm{Value}}{\mathrm{Predicted}\;\mathrm{Value}}$$

Based on the validation set, the residual standard error calculated was <2.00, which indicated no significant difference between the actual and predicted droplet size [46]. This showed that the developed model was good.

### 2.4. Optimization of the Responses

Using the desirability function method in the Design-Expert^®^ software, numerical optimization was carried out. The optimized formulation was developed based on the value of all independent variables in the range, resulting in the minimum droplet size of the nanoemulsion, as shown in Table 5.

Based on the CCD-RSM, an optimized formulation with 8.04 min (time of high shear), 4905.41 rpm (speed of high shear), and 271.82 rpm (speed of stirrer) was suggested, which would produce a droplet size of 103.71 nm. Table 6 shows the response values of the optimized KMO nanoemulsion.

### 2.5. Physicochemical Characterization of Optimized Nanoemulsion

#### 2.5.1. Mean Droplet Size, Zeta Potential and Polydispersity Index (PDI) Analysis

The mean droplet size, zeta potential and polydispersity index (PDI) of the optimized nanoemulsion containing KMO were found to be 103.97 nm, −45.4 mV and 0.312, respectively. For cosmeceutical purposes, the nanoemulsion with a droplet size ranging between 100 to 200 nm was more favorable [47]. The zeta potential measures the electrokinetic potential of a particle and was used to determine the stability of the nanoemulsion. A nanoemulsion with zeta potential values higher than +30 mV and lower than −30 mV was said to be stable [48]. PDI value was used to analyse the size distribution of particles in the nanoemulsion. The PDI value, which was near to zero, indicates a monodispersed system; meanwhile, a PDI value near to one indicates a polydispersed system (wide range of droplet sizes) [49]. Pongsumpun et al. [50] stated that a nanoemulsion system is considered monodispersed (narrow size distribution) if the PDI value is less than 0.3. The optimized nanoemulsion containing KMO was found to be polydispersed and stable, due to its PDI value being more than 0.3, and the negative value of its zeta potential. Its droplet size also makes it suitable for use in cosmeceutical applications.

#### 2.5.2. Morphology

**Figure 3:**
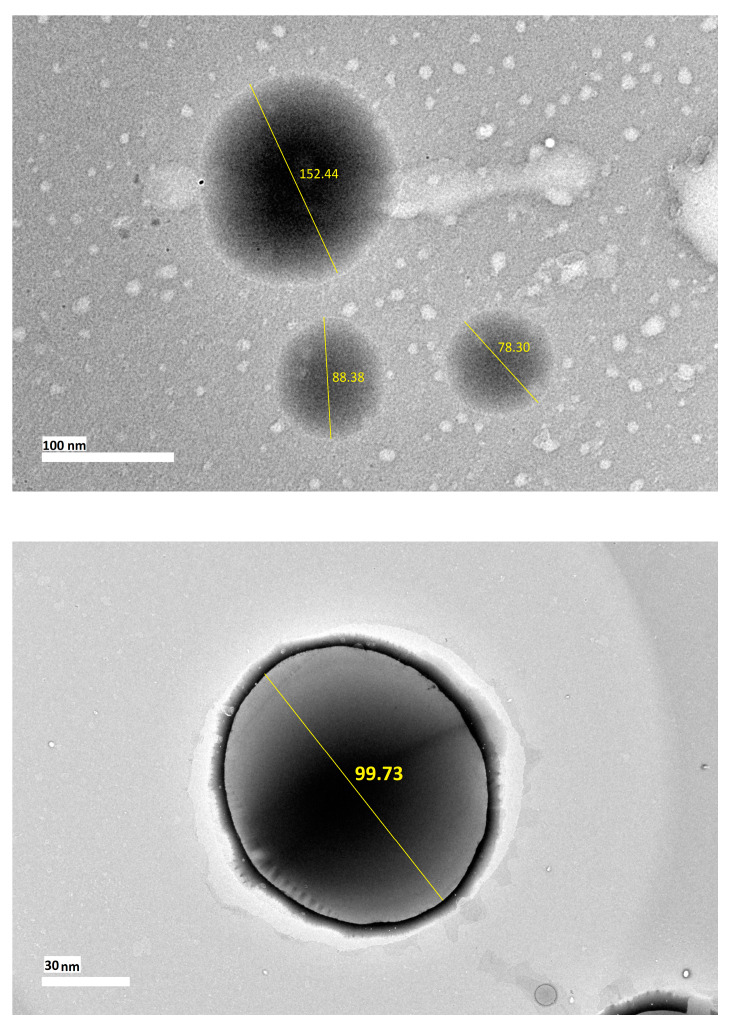
Transmission electron microscopy (TEM) images of nanoemulsion containing KMO.

Figure 3 shows the transmission electron microscopy (TEM) images of the optimized nanoemulsion containing KMO. The TEM results revealed that the optimized nanoemulsion containing KMO was a polydispersed system, with the droplet size ranging from 70 to 160 nm. The droplets were found to be in spherical shape without any aggregation present in the system. The polydispersity of the optimized nanoemulsion was correlated with the PDI value obtained from dynamic light scattering (DLS) method. Although the system was found to be polydispersed, the droplet size of the optimized nanoemulsion containing KMO was still in the nanosize range (20 to 200 nm). The main focus should be on the droplet size, in order to deliver the active ingredient efficiently. Moreover, the stability of the optimized nanoemulsion should also be taken into consideration. This is reflected through the zeta potential of the optimized nanoemulsion containing KMO (−45.4 mV), and also the physical changes or phase separation in the KMO nanoemulsion after being subjected to centrifugal force and different storage temperatures (4, 25 and 45 °C) for 90 days. The stability study revealed that the optimized nanoemulsion containing KMO was able to retain its droplet size in the nanosize range (20 to 200 nm) and a stable zeta potential (less than −40 mV), as described in the latter section of the discussion.

#### 2.5.3. pH and Conductivity Measurement

The pH of skin is around 5.5 and often a pH in the range of 4.0 to 7.0 is suitable for topical application [51,52]. The pH of the optimized nanoemulsion containing KMO was 5.75, thus making it suitable for topical application. Conductivity refers to the measurement of the free amount of water and ions, as it will respond to any and all ions present in any solution. This parameter helps to identify the type of nanoemulsion produced. The high conductivity value of optimized nanoemulsion containing KMO, which was 3.98 mS/cm, demonstrated that the aqueous phase was a continuous phase, indicating that the nanoemulsion formed was oil-in-water nanoemulsion (O/W) [53]. In the cosmetics industry, the O/W formulation is more favorable as it is less greasy after application.

#### 2.5.4. Accelerated Stability Study

Stability is one of the important parameters for nanoemulsion. A nanoemulsion should remain physically stable throughout its shelf life with no or minimal changes in the droplet size. The creaming or sedimentation (phase separation) rate of the optimized nanoemulsion (in terms of shelf life) can be determined by a centrifugation test, as the centrifugation force is equivalent to the gravitational force [54]. The optimized nanoemulsion containing KMO showed no physical changes or phase separation after being subjected to a 4000-rpm centrifugation force for 15 min.

A storage stability study was carried out by exposing optimized nanoemulsion to three different temperatures (4, 25 and 45 °C) for 90 days. Table 7 shows the physical stability of optimized nanoemulsion under different storage temperatures and also under centrifugation force.

The optimized nanoemulsion containing KMO remained a homogenous mixture and showed no physical or phase changes after 90 days under all storage temperatures.

**Figure 4:**
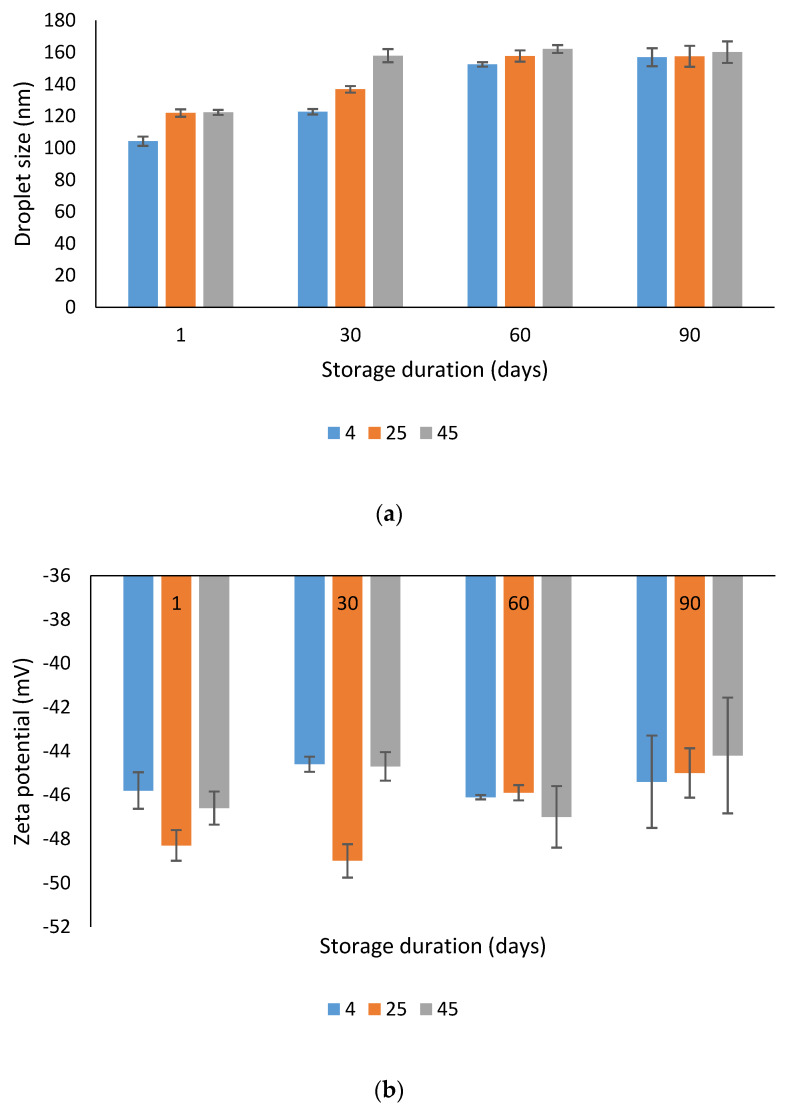

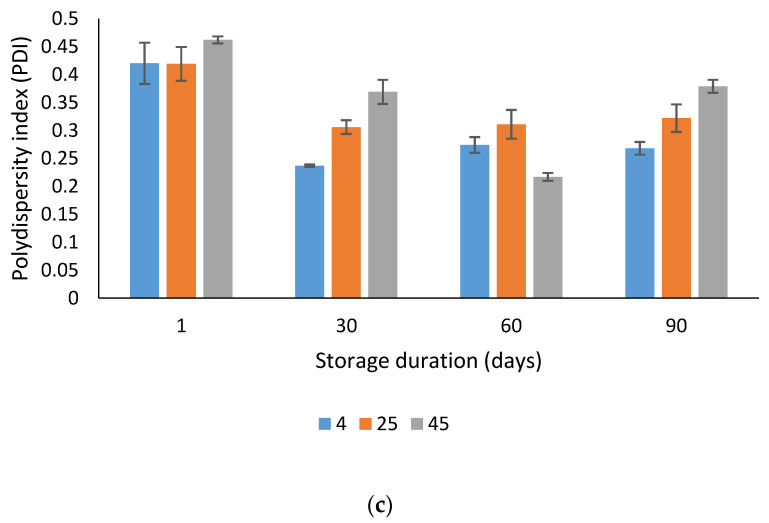
Physical stability of the optimized nanoemulsion containing KMO in terms of (**a**) droplet size; (**b**) zeta potential; (**c**) polydispersity index (PDI).

The droplet size, zeta potential and PDI values of optimized nanoemulsion for all storage temperatures were measured at day 1, 30, 60 and 90, and are presented in Figure 4.

Based on Figure 4a, the droplet size of the optimized nanoemulsion at all three different storage temperatures showed an increment. However, the nanosize of the droplet was maintained, which was between 20 to 200 nm over 90 days. Syed Azhar et al. [17] reported that the nonionic surfactant used (tween80) can prevent droplet collision, as it formed a steric barrier at the oil or water interface. This enable the optimized nanoemulsion to maintain its droplet size in the nano range.

In Figure 4b, the zeta potential of the optimized nanoemulsion containing KMO only varied in the range from −40 to −50 mV over the 90 days of the stability study. This demonstrated that the different storage temperature did not have any significant effect on the repulsion forces between droplet particles in the optimized nanoemulsion. After 90 days, the zeta potential of the optimized nanoemulsion in all storage temperatures was <−40 mV, indicating the good physical stability of the dispersed systems, as the value represents the high repulsion of droplets [55].

The PDI values [Figure 4c] at day 1 for all optimized nanoemulsions containing KMO at all storage temperatures were found to be higher than at days 30, 60 and 90. During the storage, the PDI value decreased with the accommodation and stabilization of the system. The PDI value after day 90 for all storage temperatures was less than 0.4, indicating that the optimized nanoemulsion containing KMO still exhibited properties that indicated that it was polydispersed. However, it did not exceed the initial PDI value.

**Figure 5:**
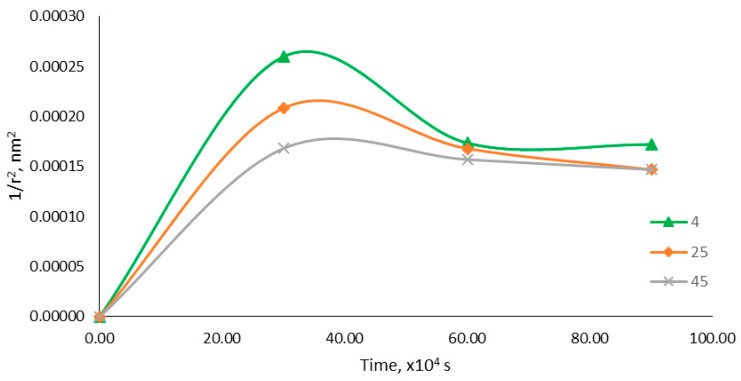
Graph of 1/r^2^ versus storage time (s).

For a better understanding, the stability study was then carried out for the coalescence rate and Ostwald ripening. Coalescence happens when the films of continuous phases are ruptured, causing two droplets to fuse together to form a larger droplet. Figure 5 shows the plotted graph of 1/r^2^ versus storage time. The increment of droplet size was said to be affected by coalescence when the plotted graph was linear. However, based on the plotted graph, all the droplet size values did not produce a linear relationship. Thus, the increment of the droplet size over time of the optimized nanoemulsion containing KMO was not affected by the coalescence rate. Sharma et al. [24] demonstrated that a small nanoemulsion droplet size can suppresses the coalescence or coagulation of nanoemulsion droplets.

**Figure 6:**
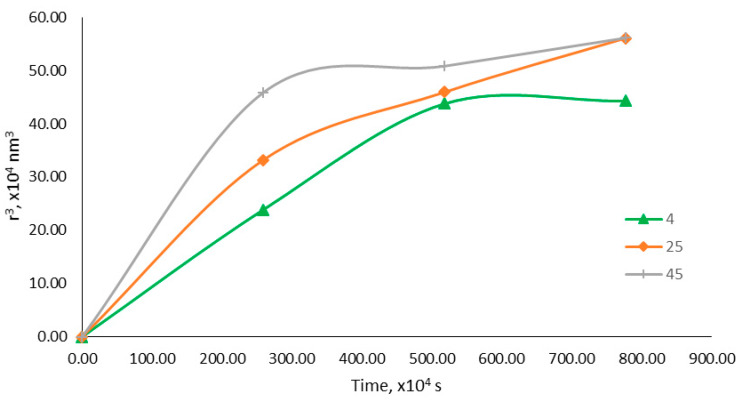
Graph of r^3^ versus storage time (s).

Ostwald ripening is a phenomenon in which small droplet particles diffuse together to form larger droplet particles. The dispersed phase in the nanoemulsion system absorbs energy from its surroundings, which leads to an increase in its kinetic energy, and the effective collision of the dispersed phase, making it possible for small droplet particles to diffuse together, forming larger droplet particles [23]. The Ostwald ripening rate can be determined by plotting r^3^ versus the storage time on a graph, as shown in Figure 6. The optimized nanoemulsion containing KMO stored in 45 °C was most affected by Ostwald ripening, followed by those at 25 °C and 4 °C. This finding correlated with the findings of Syed Azhar et al. [17], who reported that the higher the temperature is, the higher the Ostwald ripening rate is. Ribeiro et al. [48] also reported that, at 4 °C, minimal droplet size changes happened, compared to nanoemulsions stored at 25 °C and 45 °C. Mat Hadzir et al. [54] described that, at a storage temperature near to freezing temperature (4 °C), the droplets are believed to be in a nearly frozen state, which reduces the droplets’ movement or kinetic energy.

#### 2.5.5. Viscosity Determination

**Figure 7:**
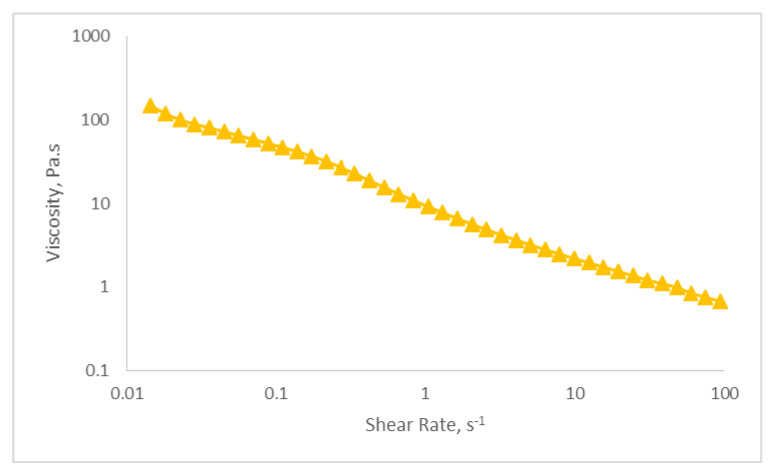
Graph of viscosity (Pa.s) versus shear rate (s^−1^).

The behavior of the optimized nanoemulsion containing KMO was studied by utilizing a steady-state test. The optimized nanoemulsion demonstrated a shear-thinning behavior (non-linear relationship) in the steady state test, as shown in Figure 7. The rheological properties of the optimized nanoemulsion can be determined by the flow behavior index (n) based on Equation (5), as shown in Table 8. The value of n is 0.366 (n < 1), which shows that the optimized nanoemulsion had shear-thinning properties. The viscosity of the optimized nanoemulsion decreased with the increasing shear rate, indicating that the optimized nanoemulsion exhibits non-Newtonian pseudo plastic fluid (n < 1). Table 8 shows the non-Newtonian pseudo plastic behavioral properties of the optimized nanoemulsion containing KMO. Samson et al. [56] found that this type of behavior was usually desired by the cosmetic and pharmaceutical industries for the formulation of products that are topically applied. When tested under high shear conditions and zero flow under gravity stress, a shear-thinning or pseudo-plastic nanoemulsion exhibits a low viscosity fluid behavior (low resistance) in relation to flow [17].

### 2.6. In Vitro Assessments of Optimized Nanoemulsion Containing KMO

#### 2.6.1. Cytotoxicity Assay

**Figure 8:**
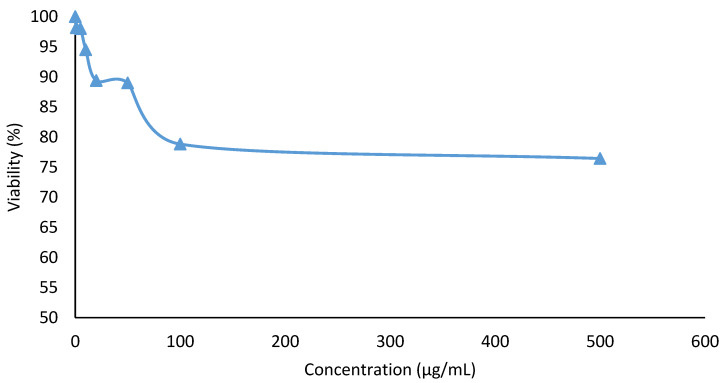
Fifty percent inhibition of cell viability (IC_50_) of optimized nanoemulsion containing KMO against 3T3 cell line.

A cytotoxicity study was done to test the safety of the optimized nanoemulsion containing KMO. A mouse embryonic fibroblast cell line (3T3) was used in this cytotoxicity study. An MTT (3-(4,5-dimethylthiazol-2-yl)-2,5-diphenyl-tetrazolium bromide) colorimetric assay was used to determine the cellular response of the 3T3 cell line on concentration-dependent cytotoxicity of the sample. The 50% inhibition of cell viability (IC_50_) of optimized nanoemulsion is shown in Figure 8.

The MTT assay indicated that the cell viability decreased with increasing concentrations of optimized nanoemulsion containing KMO. However, at the highest concentration of the optimized nanoemulsion (500 µg/mL), the cell viability was determined to be more than 50%. This means that the IC_50_ value of the optimized nanoemulsion was more than 500 µg/mL (IC_50_ > 500 µg/mL). Since the IC_50_ of the optimized nanoemulsion did not reach the highest concentration tested, this suggested that the optimized nanoemulsion containing KMO is safe and suitable to be used for cosmeceutical applications. Lajis et al. [9] also claimed that, at high concentrations (up to 500 µg/L), KMO shows very low cytotoxicity. Itharat et al. [57] stated that IC_50_ > 30 µg/mL was considered non-toxic.

#### 2.6.2. Tyrosinase Inhibition Assay

**Figure 9:**
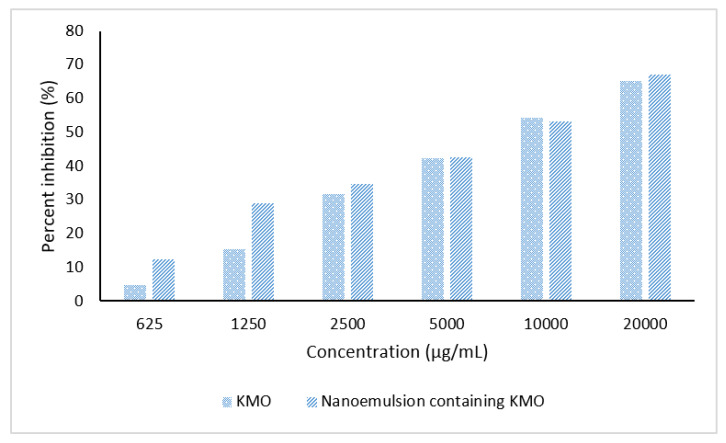
Inhibitory activity of tyrosinase enzyme by KMO and optimized nanoemulsion containing KMO.

Tyrosinase is the key enzyme that is responsible for melanin production. Excess melanin contributes to skin disorders such as hyperpigmentation, melisma and malign melanoma [4,5]. Thus, tyrosinase activity has become a major target in the development of tyrosinase inhibitors in the cosmeceuticals field [58]. A tyrosinase inhibition assay was performed by using l-tyrosine as the substrate. l-tyrosine is an amino acid, involved in the formation of dopachrome and melanin through tyrosinase-mediated oxidation. The effects of KMO itself and the optimized nanoemulsion containing KMO on tyrosinase activity were studied. As shown in Figure 9, both KMO and the optimized nanoemulsion containing KMO exhibited concentration-dependent inhibitory activity towards the tyrosinase-mediated oxidation of l-tyrosine. At all concentrations (except for 10,000 µg/mL), the inhibitory activity of the optimized nanoemulsion was superior compared to the inhibitory activity of KMO only. The inhibitory activity of nanoemulsion without KMO was also studied, but this emulsion only showed inhibitory activity at the highest concentration (20,000 µg/mL), which was 6.81%.

**Figure 10:**
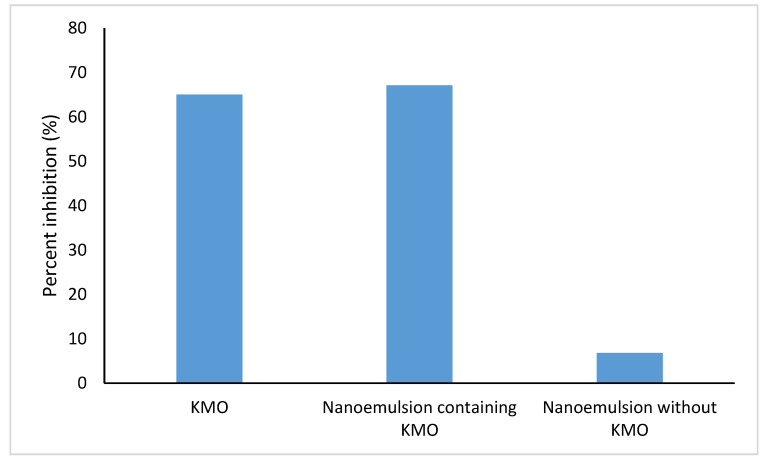
Inhibitory activity of tyrosinase enzyme at 20,000 µg/mL.

Figure 10 shows a comparison of the inhibitory activity of KMO, optimized nanoemulsion containing KMO and nanoemulsion without KMO at a concentration of 20,000 µg/mL. The optimized nanoemulsion containing KMO exhibited the highest inhibitory activity, which was 67.12%, making the optimized nanoemulsion a promising tyrosinase inhibitor for use in the cosmeceuticals field.

## 3. Materials and Methods

### 3.1. Materials

**Figure 11:**
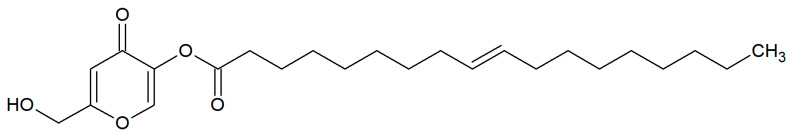
Chemical structure of kojic monooleate (KMO).

KMO (Figure 11) was synthesized according to the method used by Jumbri et al. [10]. Castor oil (CO), xanthan gum (from *Xanthomonas campestris*), potassium sorbate, mushroom tyrosinase (*Agaricus bisporus*) and l-tyrosine were purchased from Sigma-Aldrich (St. Louis, MO, USA). South African lemon essential oil (LO) was purchased from Wellness Original Ingredient (Puchong, Malaysia). Tween 80 (hydrophile-lipophile balance [HLB] 15.0) was purchased from EMD Millipore (Billerica, MA, USA). The 3T3 cell lines (from mouse embryonic fibroblast cells) were purchased commercially from the American Type Culture Collection (ATCC) (Manassas, VA, USA). Deionized water was purified using a Milli-Q water system (EMD Millipore). The chemicals used were all analytical, food, or cosmetic grade classes.

### 3.2. Preparation of KMO Nanoemulsion

For the preparation of the KMO nanoemulsion, the composition of each material was adopted from Syed Azhar et al. [17]. The oil phase was obtained by blending 10% of *w*/*w* KMO and 2.7% of *w*/*w* CO, while, for the aqueous phase, 4% of *w*/*w* Tween 80 and 1.5% of *w*/*w* xanthan gum were added to 80.8% *w*/*w* of deionized water. Both phases were heated separately up to 30 °C, while they were stirred using a magnetic stirrer to ensure the formation of a homogenous solution. Both phases were then sonicated using an ultrasonic bath sonicator (Power Sonic 405, Hwashin Technology Co., Seoul, Korea) for 16 min. The oil phase was gradually added dropwise into the aqueous phase, while it was homogenized using a high shear homogenizer (T25 digital; IKA-Werk, GmbH & Co. KG, Staufen im Breisgau, Germany). Finally, the mixture was further homogenized using an overhead stirrer (RW20 digital; IKA-Werk) for 3 h while adding 0.3% *w*/*w* LO and 0.7% *w*/*w* of potassium sorbate.

### 3.3. Experimental Design

A three-factor central composite design (CCD) was employed to determine the effect of time of the high shear (A), the speed of high shear (B), and the speed of the stirrer (C) towards the droplet size of the nanoemulsion. The duration of the stirrer was kept constant for 3 h. A summary of the independent variables and their coded levels are presented in Table 9.

The design matrix was developed using Design-Expert^®^ software (version 7.1.5; Stat Ease Inc., Minneopolis, MN, USA). A total of 20 runs was developed, and the results were statistically evaluated. Three independent variables were run at three levels for each of the individual variables. Therefore, this design involves eight factorial points, six axial points and six replicates of the center points. The effects of each variable and the interactions between variables on the outcomes can be studied independently using CCD [37]. The experiments were conducted at random to minimize the influence of the extraneous factor.

### 3.4. Statistical Analysis

Upon the completion of all runs, the analysis of variance (ANOVA) and coefficient of determination (*R*^2^) were determined to study the significant differences among the independent variables in terms of a lack of fit test. The significance of the equation parameters for each response can be achieved with a probability value (*p*-value) less than 0.05 (*p* < 0.05). In this study, the optimum levels of the processing conditions (the time of high shear, the speed of high shear, and the speed of the stirrer) were determined in order to produce a nanoemulsion with desirable responses.

### 3.5. Verification of Model

The verification of the final model was carried out in terms of a validation set in order to study the adequacy of the predicted response value. Random formulations with different conditions were considered in order to validate the model. The actual and predicted response values were used to calculate the percentage of residual standard error. The final optimum conditions suggested were also used to confirm the predicted optimum values of the model.

### 3.6. Physicochemical Characterization

#### 3.6.1. Droplet Size, Zeta Potential and Polydispersity Index (PDI) Determination

Droplet size particles, stability (zeta potential) and size distribution (PDI) in the nanoemulsion system were measured using dynamic light scattering, which scattered at an angle of 173° and a temperature of 25 °C. This process was carried out using a droplet size analyzer (Zetasizer Nano ZS90; Malvern Instruments, Malvern, UK). The measurement of droplet size was done a day after the formulations were made to ensure that the system has achieved equilibrium [34]. The required concentration of samples was obtained by diluting them with deionized water to reduce multiple scattering effects, before pouring them into a folded capillary cell (DTS1070; Malvern Instruments) [17]. The count rate was maintained between 100 and 300 kcps. The measurement was repeated at least three times and the values are reported as mean values.

#### 3.6.2. Morphology Study

The size and morphology of optimized nanoemulsion containing KMO were investigated by using Transmission Electron Microscopy (TEM) (Hitachi H7100, Tokyo, Japan). Samples were diluted in deionized water and homogenized. A Formvar-coated copper grid was placed on top of a drop of diluted sample and left at room temperature (25 ± 0.5 °C) for 3 min. The samples on the filled copper grid were then negatively stained using 2% *w*/*w* phosphotungstic acid for 2 min and were air-dried prior to analysis.

#### 3.6.3. pH and Conductivity Measurement

The pH of the optimized nanoemulsion containing KMO was determined using a Delta 320 pH meter (Mettler Toledo, Columbus, OH, USA). Before pH measurements were taken, the pH meter was calibrated with three pH standard buffer solutions (pH 4.00, 7.00 and 10.00). The pH value was determined by the direct insertion of the electrode into the sample. pH measurement is crucial to make sure that the nanoemulsion containing KMO is compatible with human skin.

Conductivity refers to the measurement of the free amount of water and ions. Conductivity measurements can be used to determine whether the nanoemulsion containing KMO is oil-in-water (O/W) or water-in-oil (W/O) nanoemulsion. The conductivity of the nanoemulsion containing KMO was determined using a conductometer (Mettler Toledo). The electrode was inserted directly into the sample before the conductivity value was measured.

#### 3.6.4. Accelerated Stability Study

Shahidan et al. [59] demonstrated that the stability of nanoemulsion is defined by the capability of the formulation (in a specific system) to maintain its physical appearance, without any phase separation or physical changes over a specific time of storage and during use. The optimized nanoemulsion containing KMO was subjected to two stability studies—stability under a centrifugation test and storage stability at different temperatures (4, 25 and 45 °C) for 90 days. The nanoemulsion containing KMO was said to be unstable if the visible appearance of creaming or phase separation occurred.

To test its stability under a centrifugation test, the optimized nanoemulsion containing KMO was kept in a centrifuge tube and was subjected to centrifugation force (EBA 200; Hettich Zentrifugen, Tuttlingen, Germany) at 4000 rpm for 15 min. The optimized nanoemulsion was then observed for any phase separation or physical change.

To determine its storage stability at different temperatures, the optimized nanoemulsion was observed in terms of its physical appearance, droplet size, zeta potential and PDI on days 1, 30, 60 and 90 of storage time.

In order to determine the factors affecting the changes in droplet sizes over time, a coalescence rate analysis was performed. All the collected data for droplet sizes in terms of storage stability for 90 days (at all storage temperatures) were analyzed and calculated using Equation (3).

(3) $$\frac{1}{r^{2}}=\frac{1}{r_{0}{}^{2}}-(\frac{8\pi }{3})\omega t$$

Based on the equation above, ***r*** is the mean radius after time, ***r_o_*** is the value at time (s) t = 0 and ω is the frequency of rupture per unit of the film surface. A graph of 1/r^2^ against the storage time (s) was plotted to evaluate the coalescence rate. A linear relationship graph was predicted for a nanoemulsion system affected by the coalescence rate.

The Lifshitz–Slesov–Wagner theory was used to determine the Ostwald ripening rate for the nanoemulsion containing KMO and the effect of storage temperature on this rate. Ostwald ripening occurred when the droplet size of the nanoemulsion system increased over a certain period of time due to the diffusion of the oil phase through the aqueous phase. The equation used to determine the Ostwald ripening rate is shown below.

(4) $$\omega =\frac{dr^{3}}{dt}=\frac{8}{9}\left[\frac{C(\infty )V_{m}D}{\rho RT}\right]$$

Based on the equation above, ω is the frequency of rupture per unit surface of the film, ***r*** is the average radius of droplets over time, ***t*** is the storage time (s), *C*(∞) is the bulk-phase solubility, ***V_m_*** is the molar volume of the internal phase, ***D*** is the diffusion coefficient of the dispersed phase in the continuous phase, ρ is the density of the dispersed phase, ***R*** is the gas constant and ***T*** is the absolute temperature. The Ostwald ripening rate was analyzed using a plotted graph of r^3^ versus the storage time (s).

#### 3.6.5. Viscosity Determination

An AR-G2 rheometer (TA instruments, New Castle, DE, USA) was used to determine the viscosity of the optimized nanoemulsion containing KMO, with 4 °C/40 mm cone and plate geometries (gap of 0.100 mm) at 25 °C. The steady-state behavior of the sample was analyzed at a controlled rate varying from 0.1 to 100 s^−1^. Prior to the measurements, the sample was allowed to stand for 10 min after being loaded in order to achieve an equilibrium state. The experimental data were fitted to the power law as in Equation (5).

η = ký^n−1^ (5)

Based on the equation above, η is the viscosity (Pa.s), ý is the shear rate (s^−1^), and k and n are the consistency index and flow behavior index, respectively.

### 3.7. In Vitro Assessments of Nanoemulsion Containing KMO

#### 3.7.1. Cytotoxicity Assay

The cytotoxic activity of the optimized nanoemulsion containing KMO was studied using an MTT (3-(4,5-dimethylthiazol-2-yl)-2,5-diphenyl-tetrazolium bromide) assay on the 3T3 mouse embryonic fibroblast cells. A cell culture with a concentration of 2 × 10^3^ cells/mL was performed and was plated (100 µL/well) onto 96-well plates. The diluted ranges of sample extracts were added to each well with identified concentrations of 500, 100, 50, 25, 10, 5 and 1 µg/mL, then further incubated for 24 h. The MTT solution was added to the cells after the samples had been incubated and then the solution was incubated again for 3 h. After the solubilisation of the purple formazan crystals using dimethyl sulfoxide (DMSO) was completed, the Optical Density (OD) of the samples were measured using an ELISA microplate reader (Los Angeles, CA, USA) at a wavelength of 570 nm. The cytotoxicity was recorded as the concentration that caused a 50% growth inhibition of the cells (IC_50_ value) using Equation (6).

(6) $$Cell\;viability\;\left(\%\right)=\frac{Absorbance\;sample\;\left(mean\right)}{Absorbance\;control\;\left(mean\right)}\times 100$$

A graph of the percentage of cell viability against the respective concentrations was then plotted.

#### 3.7.2. Tyrosinase Inhibition Assay

A tyrosinase inhibition assay was carried out as described by Cui et al. [58], with slight modifications. l-tyrosine was used as the substrate. The tyrosinase enzyme was prepared by dissolving 9.31 mg of tyrosinase enzyme in 25 mL of 50 mM phosphate buffer solution (PBS) to achieve a concentration of 1000 U/mL. The substrate was prepared by dissolving 0.036 g of l-tyrosine in 100 mL of 50 mM PBS. The sample was prepared via serial dilution, starting with the highest concentration (20000, 10000, 5000, 1250 and 625 µg/mL in PBS).

A total of 264 µL of the substrate and 50 µL of the sample were pipetted into 96-well plates and incubated for 2 min at 25 °C. Then, 6 µL of the tyrosinase enzyme was added to the mixture and further incubated for 10 min at 25 °C. The absorbance was measured at 492 nm using an ELISA microplate reader (Labomed, model UVD-2950, Los Angeles, CA, USA). For the control, the sample was replaced with 50 µL of PBS. The tyrosinase inhibitory activity was calculated as in Equation (7):

(7) $$Percent\;inhibition\left(\%\right)=\frac{A-B}{A}\times 100\;$$

where A and B are the absorbance of the control and the sample, respectively. Each experiment was carried out in triplicate.

## 4. Conclusions

This study indicates that our optimized nanoemulsion formulation containing KMO and using RSM, is an excellent approach for investigating the effects of the time of high shear, the speed of high shear and the speed of the stirrer on the response (droplet size). ANOVA demonstrated the fitness of the model, with *F*-values (148.79) and *p*-values (*p* < 0.0001) that showed an insignificant lack of fit, with a high coefficient of determination *R*^2^ = 0.9933. The model was verified further by testing some random formulations under different conditions. The optimized condition for the formulation of KMO nanoemulsion was 8.04 min (time of high shear), 4905.42 rpm (speed of high shear), and 271.77 rpm (speed of stirrer), which resulted in a 103.97 nm droplet size. The physicochemical characterization showed that the nanoemulsion containing KMO was in the nanosize range and had a zeta potential of −45.4 mV and a PDI of 0.312, indicating that the nanoemulsion produced was stable and polydispersed. A morphology study using TEM revealed that the oil droplets in the optimized nanoemulsion containing KMO were spherical in shape, without any aggregation present in the system. The pH of the optimized nanoemulsion was 5.75, which makes it compatible with skin pH (4.0–7.0) and its conductivity was high, indicating that the optimized nanoemulsion produced was an oil-in-water nanoemulsion. The optimized nanoemulsion containing KMO remained stable (no phase separation was observed) under a centrifugation test and during storage at 4, 25 and 45 °C over 90 days. Finally, a cytotoxicity assay showed that our optimized nanoemulsion was less toxic than others, with IC_50_ > 500 μg/mL, and that it can inhibit 67.12% of tyrosinase activity.

## Figures and Tables

**Table 1:** Experimental data actual and predicted values of the droplet size of the kojic monooleate (KMO) nanoemulsion.

Standard Order	Run Order	Independent Variables			Droplet Size (nm)	
		Time of High Shear (min)	Speed of High Shear (rpm)	Speed of Stirrer (rpm)	Actual	Predicted
1	1	8.04	4905.40	220.27	118.14	119.40
2	16	16.96	4905.40	220.27	145.50	145.22
3	3	8.04	6094.60	220.27	132.47	132.11
4	20	16.96	6094.60	220.27	118.30	117.24
5	13	8.04	4905.40	279.73	103.16	104.08
6	11	16.96	4905.40	279.73	148.00	148.22
7	10	8.04	6094.60	279.73	116.80	116.94
8	15	16.96	6094.60	279.73	121.79	120.39
9	17	5.00	5500.00	250.00	116.03	114.80
10	18	20.00	5500.00	250.00	138.00	139.42
11	8	12.50	4500.00	250.00	128.00	126.67
12	12	12.50	6500.00	250.00	112.43	113.96
13	9	12.50	5500.00	200.00	131.50	131.70
15	19	12.50	5500.00	250.00	113.00	111.82
16	2	12.50	5500.00	250.00	112.67	111.82
17	4	12.50	5500.00	250.00	111.45	111.82
18	14	12.50	5500.00	250.00	110.73	111.82
19	5	12.50	5500.00	250.00	112.60	111.82
20	6	12.50	5500.00	250.00	110.53	111.82

**Table 2:** ANOVA results for the effect of all independent variables.

Source	Sum of Squares	df	Mean Square	*F*-Value	*p*-Value	Significant
Model	2850.06	9	316.67	148.79	<0.0001	significant
A	731.78	1	731.78	343.82	<0.0001	
B	195.15	1	195.15	91.69	<0.0001	
C	82.68	1	82.68	38.85	0.0002	
AB	827.84	1	827.84	388.95	<0.0001	
AC	167.81	1	167.81	78.85	<0.0001	
BC	0.011	1	0.011	5.286 × 10^−3^	0.9436	
A^2^	400.66	1	400.66	188.25	<0.0001	
B^2^	123.53	1	123.53	58.04	<0.0001	
C^2^	228.97	1	228.97	107.58	<0.0001	
Residual	19.16	9	2.13			
Lack of Fit	13.44	4	3.36	2.94	0.1337	not significant
Pure Error	5.71	5	1.14			
Corrected Total	2869.22	18				

**Table 3:** Regression coefficient results for the final reduced model.

**SD**	1.46	***R*** ^2^	0.9933
**Mean**	121.11	**Adjusted *R*^2^**	0.9866
**CV%**	1.20	**Predicted *R*^2^**	0.9494
**PRESS**	145.07	**Adequate Precision**	41.710

**Table 4:** Validation set for verification of the final model obtained.

Independent Variable			Droplet Size (nm)		RSE (%)
A (min)	B (rpm)	C (rpm)	Actual	Predicted	
10.00	6000	250.00	115.31	113.16	1.90
12.00	5600	225.00	116.08	117.39	1.16
10.00	5800	300.00	117.82	116.51	1.12
13.00	5600	250.00	113.06	111.97	0.97

**Table 5:** Constraints of numerical optimization.

Constraints	Goal	Lower Limit	Upper Limit
Time of high shear (A)	In range	8.04	16.96
Speed of high shear (B)	In range	4905.40	6094.60
Speed of stirrer (C)	In range	220.27	279.73
Droplet size	Minimize	102.60	183.02

**Table 6:** Optimum formulation of KMO nanoemulsion.

Independent Variable			Droplet Size (nm)		Desirability
A (min)	B (rpm)	C (rpm)	Actual	Predicted	
8.04	4905.41	271.82	103.97	103.71	0.988

**Table 7:** Physical stability of optimized nanoemulsion containing KMO under different storage temperatures (4, 25 and 45 °C) and centrifugation test.

Storage Temperature (°C)	Storage Stability (days)				Centrifugation
	1	30	60	90	
4	/	/	/	/	
25	/	/	/	/	/
45	/	/	/	/	

/ = stable/no physical change.

**Table 8:** Flow behavior indices (n), consistency coefficients (k), and regression coefficients (R^2^) of the optimized nanoemulsion containing KMO.

Sample	k	n	R^2^
Optimized nanoemulsion containing KMO	10.227	0.366	0.996

**Table 9:** Summary of independent variables and their coded levels.

Independent Variables	Unit	Coded Level				
		−2	−1	0	+1	+2
A	min	5.00	8.04	12.50	16.96	20.00
B	rpm	4500.00	4905.40	5500.00	6094.60	6500.00
C	rpm	200.00	220.27	250.00	279.73	300.00
